# Predictors of Success for Pulmonary Vein Isolation With Pulsed-field Ablation Using a Variable-loop Catheter With 3D Mapping Integration: Complete 12-month Outcomes From inspIRE

**DOI:** 10.1161/CIRCEP.123.012667

**Published:** 2024-04-24

**Authors:** Tom De Potter, Massimo Grimaldi, Mattias Duytschaever, Ante Anic, Johan Vijgen, Petr Neuzil, Hugo Van Herendael, Atul Verma, Allan Skanes, Daniel Scherr, Helmut Pürerfellner, Gediminas Rackauskas, Pierre Jais, Vivek Y. Reddy

**Affiliations:** 1 OLV Hospital, Dienst Cardiologie, Aalst, Belgium (T.D.P.).; 2 Ospedale Generale Regionale “F. Miulli” UOC Cardiologia, Bari, Italy (M.G.).; 3 AZ Sint Jan, Brugge, Belgium (M.D.).; 4 University Hospital Center Split, Split, Croatia (A.A.).; 5 Jessa Hospitals, Hasselt, Belgium (J.V.).; 6 Department of Cardiology, Na Homolce Hospital, Prague, Czech Republic (P.N.).; 7 Ziekenhuis Oost, Genk, Belgium (H.V.H.).; 8 McGill University Health Center, Montréal, Canada (A.V.).; 9 University of Western Ontario, London, Canada (A.S.).; 10 Medical University Graz, Graz, Austria (D.S.).; 11 Ordensklinikum Linz GmbH, Linz, Austria (H.P.).; 12 Centre for Cardiology & Angiology, Department of Cardiovascular Diseases, Vilnius University, Lithuania (G.R.).; 13 IHU LIRYC ANR-10-IAHU-04, Centre Hospitalier Universitaire Bordeaux, Bordeaux University, Bordeaux, France (P.J.).; 14 Helmsley Electrophysiology Center, Mount Sinai Fuster Heart Hospital, New York, NY; Department of Cardiology, Na Homolce Hospital, Prague, Czech Republic (V.Y.R.).

**Keywords:** atrial fibrillation, catheters, electroporation, pulmonary veins

## Abstract

**BACKGROUND:**

We previously presented the safety and early efficacy of the inspIRE study (Study for Treatment of Paroxysmal Atrial Fibrillation [PAF] by Pulsed-field Ablation [PFA] System With Irreversible Electroporation [IRE]). With the study’s conclusion, we report the outcomes of the full pivotal study cohort, with an additional analysis of predictors of success.

**METHODS:**

InspIRE was a prospective, multicenter, single-arm clinical trial of drug-refractory paroxysmal atrial fibrillation. Pulmonary vein isolation was performed with a variable-loop circular catheter integrated with a 3-dimensional mapping system. Follow-up with 24-hour Holter was at 3, 6, and 12 months, as well as remote rhythm monitoring: weekly from 3 to 5 months, monthly from 6 to 12 months, and for symptoms. The primary effectiveness end point (PEE) was acute pulmonary vein isolation plus freedom from any atrial arrhythmia at 12 months. Additional subanalyses report predictors of PEE success.

**RESULTS:**

The patient cohort included 186 patients: aged 59±10 years, female 30%, and CHA_2_DS_2_-VASc 1.3±1.2. The previously reported primary adverse event rate was 0%. One serious procedure-related adverse event, urinary retention, was reported. The PEE was achieved in 75.6% (95% CI, 69.5%–81.8%). The clinical success of freedom from symptomatic recurrence was 81.7% (95% CI, 76.1%–87.2%). Simulating a monitoring method used in standard real-world practice (without protocol-driven remote rhythm monitoring), this translates to a freedom from all and symptomatic recurrence of 85.8% (95% CI, 80.8%–90.9%) or 94.0% (95% CI, 90.6%–97.5%), respectively. Multivariate analyses revealed that left ventricular ejection fraction ≥60% (adjusted odds ratio, 0.30) and patients receiving ≥48 PFA applications (adjusted odds ratio, 0.28) were independent predictors of PEE success. Moreover, PEE success was 79.2% in patients who received ≥12 PFA applications per vein compared with 57.1% in patients receiving fewer PFA applications.

**CONCLUSIONS:**

The inspIRE study confirms the safety and effectiveness of pulmonary vein isolation using the novel 3-dimensional mapping integrated circular loop catheter. An optimal number of PFA applications (≥48 total or ≥12 per vein) resulted in an improved 1-year success rate of ≈80%.

**REGISTRATION:**

URL: https://www.clinicaltrials.gov; Unique identifier: NCT04524364

**WHAT IS KNOWN?**

- Growing evidence on novel pulsed-field ablation (PFA) technology shows safety benefits versus conventional radiofrequency ablation in both preclinical and clinical models.
- Preliminary results from the inspIRE study (Study for Treatment of Paroxysmal Atrial Fibrillation [PAF] by PFA System With Irreversible Electroporation) demonstrated favorable safety and effectiveness of PAF ablation using a novel fully integrated biphasic PFA system with a variable-loop circular catheter in combination with a multichannel PFA generator and a 3-dimensional mapping system (PFA Platform).

**WHAT THE STUDY ADDS**

- The final results of the inspIRE study confirm the favorable safety profile of the PFA system for the entire 12-month follow-up.
- Twelve-month effectiveness was comparable with multicenter experience with radiofrequency ablation technologies, and post hoc analysis showed a higher effectiveness rate in patients with an optimal number of PFA applications, demonstrating the first clinical demonstration of energy dosing for efficacy outcomes.

The promising potential of pulsed-field ablation (PFA) to replace conventional thermal modalities in cardiac arrhythmia treatment has prompted the development of various new ablation systems.^1-3^ Several recent studies, from large multicenter interventional trials to single-center case series, shed light on the safety and effectiveness of PFA for treating atrial fibrillation (AF). However, further research and analyses are needed in this early stage of innovation to understand how electrophysiologists and patients in real-world practice might benefit from this new technology.^4-9^

The inspIRE study (Study for Treatment of Paroxysmal AF by PFA System With Irreversible Electroporation) investigated the safety and effectiveness of paroxysmal AF ablation using a new biphasic PFA system with a variable-loop circular catheter (VLCC) integrated with the multichannel PFA generator and a 3-dimensional (3D) mapping system. Using an adaptive study design, early success was declared based on planned interim analyses when all patients in the pivotal cohort reached 3-month follow-up and 83 patients reached 12-month follow-up. These interim results have been reported previously, showing zero primary adverse events (AEs) or esophageal lesions of thermal origin, along with short procedure time (70.1 minutes), transpired PFA time (26.7 minutes), and fluoroscopy time (7.8 minutes).^10^

With the completion of the inspIRE trial, we now report the long-term outcomes of the full pivotal study cohort of 186 patients, including additional subanalyses that reflect standard-of-care rhythm monitoring and varied anesthetic approaches. Furthermore, predictors of long-term effectiveness and pulmonary vein (PV) reconnection analysis at repeat procedures were also included.

## METHODS

### Study Design and Population

The data that support the findings of this study are available upon request submitted through the Yale Open Data Access Project site at http://yoda.yale.edu. Full details on the inspIRE study (ClinicalTrials.gov identifier: NCT04524364) were published in the aforementioned publication.^10^ Briefly, this was a prospective, multicenter, single-arm clinical trial conducted in 13 institutions across Canada and Europe from March 2021 to May 2022 (Table S1). Adult patients (aged ≤75 years) with drug-refractory (ie, failed ≥1 class I–IV antiarrhythmic drug) symptomatic paroxysmal AF underwent first-time PV isolation (PVI) and were followed up to 12 months after the procedure. A complete description of inclusion and exclusion criteria is included in Table S2. The feasibility phase (Wave I) was conducted in Europe only and enrolled a small set of patients to assess initial safety and effectiveness. The main pivotal study phase (Wave II) enrolled patients in Europe and Canada, where study success was defined as meeting primary safety and effectiveness end points against predefined performance goals. The study was approved by national authorities and ethics committees, and all patients provided written informed consent.

### Study Procedure and Follow-Up

The study device and procedure have been described previously.^10^ Briefly, PVI was performed under sedation or general anesthesia with a multielectrode, irrigated VLCC (the Varipulse Catheter) in combination with the Trupulse Generator and the Carto3 Mapping System (Biosense Webster, Inc, Irvine, CA). After anatomic mapping (protocol-driven) and voltage mapping (per institution practice) with a diagnostic catheter or the VLCC, lesions were created according to workflow recommendations (maximum energy setting and applying ≥12 applications per PV [ie, 4 sets of 3 consecutive applications; per patient, this is the equivalent of 48 applications or 36 applications if right or left common veins were treated as 1]). The entrance block was confirmed by elimination of the signal upon adenosine/isoproterenol challenge, without a waiting period. Antiarrhythmic drug management during the follow-up period was at the discretion of the investigator.

Monitoring of atrial arrhythmia recurrence during the follow-up evaluation period included remote rhythm monitoring (weekly between months 3 and 5, monthly between months 6 and 12, and following any symptomatic episodes, recorded for a duration of 1 minute) and 24-hour Holter monitoring (at months 3, 6, and 12). ECG monitoring was conducted at preprocedure, predischarge, and the months 1, 3, 6, and 12 follow-up visits. A core laboratory independently evaluated all recurrence recordings. For patients undergoing repeat procedures, mapping of the left atrium was performed, followed by assessment for PVI and identification of any arrhythmias requiring ablation with a commercially available ablation system.

### Safety and Effectiveness Outcomes

The primary safety end point was described in detail in the interim study publication.^10^ Here, we present the device- and procedure-related serious AEs for all Wave II patients completing the study period. All primary safety events per-protocol definition were adjudicated by an independent Clinical Event Committee.

The primary effectiveness end point was based on 12-month freedom from documented episodes of asymptomatic or symptomatic atrial arrhythmia (AF, atrial flutter, or atrial tachycardia) that lasted ≥30 seconds based on electrocardiographic data after a 3-month blanking period. Failure to confirm the entrance block in all PVs was also considered a long-term effectiveness failure. Clinical success was based on 12-month freedom from documented symptomatic atrial arrhythmia recurrence.

To put into perspective the 12-month effectiveness data compared with outcomes observed in legacy AF trials and current real-world clinical practice, a post hoc analysis of effectiveness evaluated without protocol-driven remote arrhythmia monitoring was performed using modeling of previously reported study data.^8,11,12^ Also, predictors of primary effectiveness were analyzed, taking into consideration patient- and procedure-related factors.

### Repeat Ablation Analysis

For repeat ablation procedures where analyzable electroanatomical mapping system files were available, a retrospective analysis of the CARTO files was performed manually by identifying PV reconnections (which were the sites of successful re-isolation using radiofrequency energy within the PV) and assigning a location to each of them. For analysis, all PVs were divided into 4 quadrants (anterior-superior; anterior-inferior; posterior-inferior; and posterior-superior) to categorize the location for PV reconnection.

### Statistical Methods

All analyses are based on the Wave II main study cohort only. Primary effectiveness, clinical success, and repeat ablation results are summarized with Kaplan-Meier curves and 1-year survival estimates with 95% CIs.

Logistic regression modeling was performed to identify potential risk factors associated with primary effectiveness failure. Univariate analysis was performed initially to evaluate the association with patient demographics, baseline characteristics, and procedural parameters. Variables with statistically significant associations observed at *P*<0.20 from the univariate analysis were then considered for multivariate modeling. Variables with high multicollinearity were excluded from the multivariate modeling.

The primary effectiveness end point was compared between different procedural workflows among patients who had 4 veins (right superior PV, right inferior PV, left superior PV, and left inferior PV; excluding patients with common veins) ablated. Subjects with repeat ablation of PVs using nonstudy catheters during the blanking period were excluded. The patients who received ≥48 PFA applications were compared against those who received <48 ablations. Additionally, patients who received ≥12 PFA applications per vein were compared against patients who had ≥1 vein and received <12 PFA applications. Fisher’s exact test was used to identify associations between workflow and the primary effectiveness end point. Multivariable analysis, including the study center as a repeated measure using a general estimating equation approach, was performed as well.

To compare procedural efficiency between different anesthetic settings, the Kruskal-Wallis test was used to compare differences in procedure, fluoroscopy, left atrial dwell, and mapping times between Wave II Main study patients treated with conscious sedation compared with general anesthesia.

All statistical analyses were performed using SAS 9.4 or SAS Studio 3.8 (SAS Institute, Inc, Cary, NC) or R (version 4.2.0; R Foundation for Statistical Computing, Vienna, Austria).

## RESULTS

### Study Overview

Patient enrollment and patient characteristics for the primary safety and effectiveness results were reported in the interim analysis publication.^10^ The Wave II per-protocol population consisted of 186 patients, with 184 patients with a known primary effectiveness outcome and 182 completing the study.

Patients were generally young (mean age, 59.4 years), the mean CHA_2_DS_2_-VASc score was 1.3, and the most common comorbidity was hypertension (46.8%). The overall compliance for Holter and remote rhythm monitoring was 90.9% and 75.9%, respectively.

### Safety and Effectiveness Outcomes

The previously reported primary AE rate was 0%. One serious procedure-related AE, urinary retention, was reported and resolved completely.

**Figure 1.**
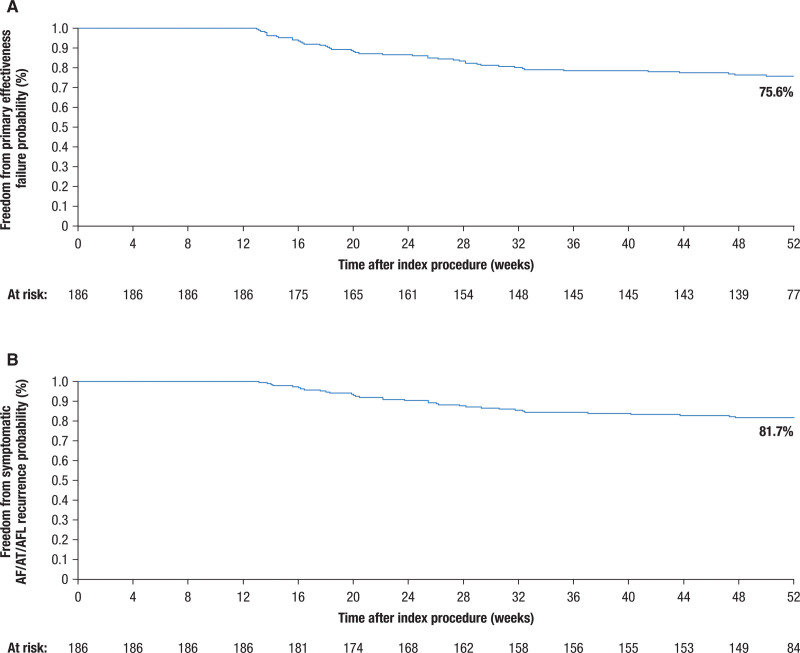
**Effectiveness analysis in the per-protocol population. A**, The primary effectiveness end point, which is 12-month freedom from AF/AT/AFL asymptomatic/symptomatic recurrences, and (**B**) clinical success, which is 12-month freedom from AF/AT/AFL symptomatic recurrences. AF indicates atrial fibrillation; AFL, atrial flutter; and AT, atrial tachycardia.

The primary effectiveness end point of the full per-protocol cohort was 75.6% (95% CI, 69.5%–81.8%; Figure 1A). Clinical success of freedom from symptomatic atrial arrhythmia recurrence was 81.7% (95% CI, 76.1%–87.2%; Figure 1B). Simulating the rhythm monitoring methods used in real-world practice (without protocol-driven remote arrhythmia monitoring), modeling the study outcome translated to freedom from all recurrence and freedom from symptomatic recurrence of 85.8% (95% CI, 80.8%–90.9%) and 94.0% (95% CI, 90.6%–97.5%), respectively (Figure S1). Twelve-month freedom from repeat ablation after the blanking period for the study of arrhythmia was 92.4% (2-sided 95% CI, 88.5%–96.2%).

### Predictors of Ablation Outcome

**Figure 2.**
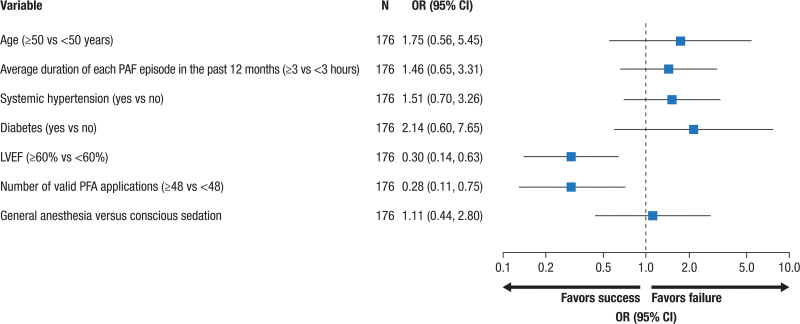
**Predictors of primary effectiveness in Wave II.** LVEF indicates left ventricular ejection fraction; OR, odds ratio; PAF, paroxysmal atrial fibrillation; and PFA, pulsed-field ablation.

**Figure 3.**
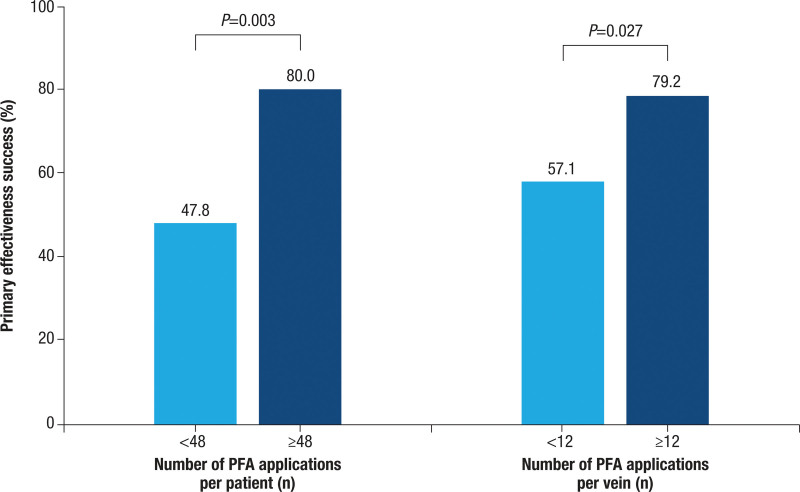
**Primary effectiveness success rate versus number of PFA applications per patient and per vein (post hoc analysis of Wave II cohort, n=158).** PFA indicates pulsed-field ablation.

The Table summarizes factors associated with primary effectiveness. Multivariate logistic regression analysis showed that the left ventricular ejection fraction (≥60% versus <60%; adjusted odds ratio, 0.30 [95% CI, 0.14–0.63]) and the number of valid PFA applications around the PV (≥48 versus <48; adjusted odds ratio, 0.28 [95% CI, 0.11–0.75]) were independent predictors of long-term effectiveness failures, with more than double the likelihood of 12-month success and odds ratios toward failure of 0.30 and 0.28, respectively (*P*<0.05; Figure 2). This suggests that there was an ≈70% reduction in the odds of primary effectiveness failure. Among the 158 patients from Wave II with 4 major veins isolated and a known primary effectiveness outcome, those with ≥48 total PFA applications or ≥12 PFA applications per vein had significantly higher primary effectiveness success rates compared with patients who received a lower number of PFA applications (80.0% versus 47.8% for ≥48 versus <48 applications per patient, respectively, *P*=0.003; 79.2% versus 57.1% for ≥12 versus <12 applications per vein, respectively, *P*=0.027; Figure 3). The general estimating equation model yielded identical independent predictors with similar odds ratios (Figure S2; Table S3).

**Table.**
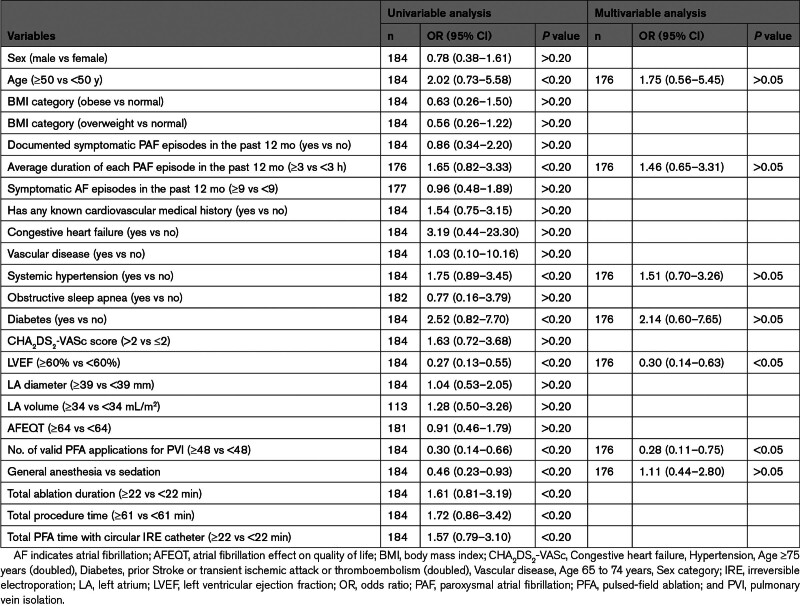
Predictors of Primary Effectiveness End Point: Logistic Regression Analysis of Wave II Cohort

**Figure 4.**
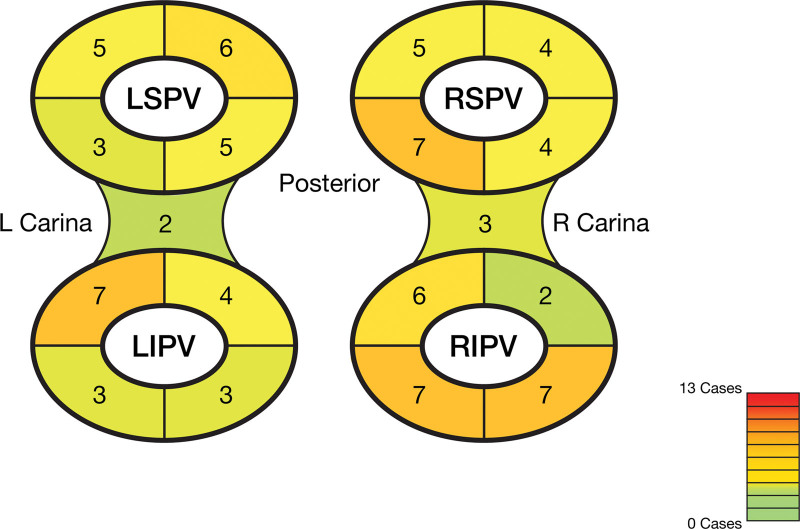
**Frequency of PV reconnection by location at repeat procedure.** LIPV indicates left inferior pulmonary vein; LSPV, left superior pulmonary vein; PV, pulmonary vein; RIPV, right inferior pulmonary vein; and RSPV, right superior pulmonary vein.

A total of 14 of 186 (7.5%) patients underwent repeat ablation procedures during the study period. Taken together, PV reconnections were noted in 37 of 51 (72.5%) veins. Of these, 13 repeat ablation procedures had electroanatomical mapping data accessible for analysis. There was no statistical difference in PV locations where reconnections occurred. Numerically, the most frequent points of reconnection were the inferior aspect of the right inferior PV (Figure 4). Noticeably, both the left and right carinas required less ablation than any vein.

About 30% of the procedures among Wave II patients were performed under conscious sedation. The use of the sedation method did not have an effect on treatment outcome after adjusting for other factors (Table), although sedation resulted in slightly higher procedure time (+4.24 minutes) and fluoroscopy time (+0.32 minutes) compared with general anesthesia. There was no difference observed in left atrial dwelling time or mapping time (Table S4).

## DISCUSSION

The final results from the inspIRE study of PFA using the VLCC with PFA generator and 3D mapping system demonstrated a primary effectiveness rate of 75.6% and a clinical success rate of 81.7% at 12-month follow-up. The simulated primary effectiveness and clinical success rate based on standard-of-care monitoring were 85.8% and 94.0%, respectively. The optimal number of PFA applications (≥48 total or ≥12 per vein) resulted in improved 1-year success of ≈80%.

**Figure 5.**
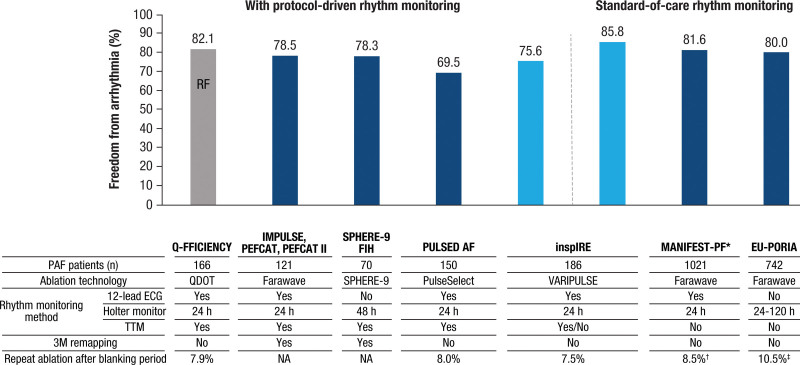
**Freedom from atrial arrhythmia at 1 year in patients with paroxysmal AF reported among recently published multicenter studies of pulsed-field ablation.** AF indicates atrial fibrillation; AFL, atrial flutter; AT, atrial tachycardia; EU-PORIA, EUropean real-world outcomes with Pulsed field ablatiOn in patients with symptomatic atRIAl fibrillation; FIH, first in human; IMPULSE, A Safety and Feasibility Study of the IOWA Approach Endocardial Ablation System to Treat Atrial Fibrillation; inspIRE, Study for Treatment of PAF by Pulsed-field Ablation System With Irreversible Electroporation; MANIFEST-PF, Multi-National Survey on the Methods, Efficacy, and Safety on the Post-Approval Clinical Use of Pulsed Field Ablation; PAF, paroxysmal atrial fibrillation; PEFCAT, A Safety and Feasibility Study of the FARAPULSE Endocardial Ablation System to Treat Paroxysmal Atrial Fibrillation; PEFCAT II, Expanded Safety and Feasibility Study of the FARAPULSE Endocardial Multi Ablation System to Treat Paroxysmal Atrial Fibrillation; PF, pulsed field; Q-FFICIENCY, Evaluation of QDOT MICRO Catheter for Pulmonary Vein Isolation in Subjects With Paroxysmal Atrial Fibrillation; RF, radiofrequency; SPHERE-9 FIH, Safety and Performance Assessment of the Sphere-9 Catheter and the Affera Mapping and RF/PF Ablation System to Treat Atrial Fibrillation; and TTM, transtelephonic monitoring. *MANIFEST reported single-procedure freedom from AF/AT/AFL. †MANIFEST repeat procedure rate calculated based on a study manuscript reporting 87 repeat ablations among 1021 patients with PAF. ‡EU-PORIA repeat procedure rate calculated based on a study manuscript reporting 78 repeat ablations among 742 patients with PAF.

These results add to the body of evidence that PFA can selectively target cell death with short energy delivery, isolating the PVs to a similar effect as thermal ablation, as demonstrated in a recent randomized controlled study.^13^ Compared with the early experience of thermal ablations, the novel PFA platform was safer and allowed for more efficient procedures. In the inspIRE pivotal trial, procedures lasted an average of 70.1 minutes and yielded zero major complications (primary AEs); >90% of patients were free from repeat procedures at 12 months. These effectiveness results are consistent with the already-concluded clinical trials and postapproval multicenter studies of other PFA catheters and systems (Figure 5).^5,7-9,14,15^ Although safety and effectiveness outcomes appear similar among various PFA devices, a notable difference between the PFA platform integrated with 3D electroanatomical mapping compared with those without such integration is the lower fluoroscopy time reported (4–8 minutes^7,10^ versus >20 minutes^9,13^ fluoroscopy time), partly attributable to the integrated intracardiac ultrasound system, which enables real-time visualization of catheters. The integrated 3D electroanatomical mapping system also provides information about electrode-tissue contact, which is known to be important for quality lesion formation.^3, 16^

Another salient finding from this report is the importance of an optimal number of PFA applications to improve long-term clinical effectiveness. While not mandated by the protocol, workflow recommendations were provided to investigators to use the maximum energy setting to apply ≥12 applications per PV. A workflow that delivered less than this recommendation more than doubled the likelihood of arrhythmia recurrence. In fact, 12-month effectiveness improved to 80% in patients with the optimal number of PFA applications. The clinical finding of this application delivery threshold for efficacy aligns well with preclinical evidence that established effective nominal dose parameters,^17^ where 12 applications per PV were required to render lesion contiguity and transmurality throughout the atria. Given the cumulative impact of successive PFA applications on tissue permeability and lesion depth,^18^ it is plausible that, with this specific pulse recipe, employing 12 applications per vein is optimal for achieving the required depth for durable PVI.^3^ Whether this observation translates to real-world practice will need further evaluation. Other factors impacting efficacy included older age and certain baseline comorbidities (eg, high left ventricular ejection fraction, hypertension, and diabetes).

In the small group of patients requiring repeat procedures, the chances of the occurrence of PV reconnection seem to be similarly distributed across different PV locations (ie, PV anatomy did not seem to play a role), including the carinas, which are generally more prone to PV reconnection with thermal ablation. This observation suggested that the recommended number of PFA applications is sufficient for various tissue thicknesses and that additional factors may have contributed to the observed reconnections.

In our study, anesthesia use varied at the site operator’s discretion, with just a slight impact on procedure time. A European single-center case series described the feasibility of PVI with VLCC using deep sedation, which demonstrated a good safety profile with positive patient reported satisfaction.^19^ According to this report, the sedation protocol was effective in pain management across these patients while mitigating the risk of diaphragm/muscle contraction and cough. There is no standardized deep-sedation protocol with PFA; further research in this area is needed.

### Limitations

This trial involves an exclusive group of patients undergoing procedures with the investigational device using a strict protocol. The impact of baseline and procedural factors on the effectiveness outcome will need to be reassessed in larger, heterogeneous populations. The comparison with contemporary ablation technologies was indirect, as the current trial is a single-arm study.

### Conclusions

The inspIRE study results demonstrated a strong safety profile and near 80% long-term effectiveness with optimal PFA application and minimal fluoroscopy using the novel VLCC in combination with a PFA generator and 3D mapping system. While the inspIRE study shows promising results, efficiency and effectiveness will likely continue to improve with broader adoption and experience as it is introduced into routine clinical practice.

## ARTICLE INFORMATION

### Acknowledgments

The authors would like to express their deepest gratitude to all investigators, including coinvestigators and guest operating investigators. They wish to thank the following individuals for their support in trial design, execution, statistical analysis, medical writing, and additional value to developing the manuscript: Tara Gomez, Eric Byun, Nathalie Macours, Carmen Rousseeuw, Sarah Rabau, Lee Ming Boo, Christina Kaneko, Jaclyn Alcazar, Yanmin Wang, Tiffany Tan, Guixia Huang, Stephen Hynes, and Puneet Jatana. Michelle Hughes, BSc, with Lumanity Communications Inc. (Yardley, PA), provided medical writing and editorial support, in accordance with Good Publication Practice guidelines, which were funded by Biosense Webster, Inc, under direction of the authors.

### Sources of Funding

This study was funded by Biosense Webster, Inc.

### Disclosures

Dr De Potter has received consulting fees and honoraria for lectures and presentations from Biosense Webster and Adagio Medical (all payments were directed to the institution). Dr Grimaldi has an unrelated patent agreement with Biosense Webster, Inc. Dr Duytschaever has served on the speakers’ bureau and as a consultant for Biosense Webster, Inc, and has received research support from Biosense Webster, Inc. Dr Anic has received consulting fees and has contracted research with Farapulse, Boston Scientific, Galaxy Medical, and Biosense Webster, Inc. Dr Vijgen has received grant support from Biosense Webster, Inc. Dr Neuzil has received grant support from Biosense Webster, Inc. Dr Van Herendael has received support from Biosense Webster, Inc, for congress-related activities. Dr Verma has received grants from Biosense Webster, Inc, Medtronic, Bayer, and Biotronik; has received consulting fees from Biosense Webster, Inc, Medtronic, Adagio Medical, Galaxy Medical, Ablacon, and Thermedical; and has received honoraria for lectures from Biosense Webster, Inc, and Medtronic. Dr Skanes has served on the speakers’ bureau for Biosense Webster, Inc, and has received research support from Biosense Webster, Inc. Dr Scherr has received grant support from Biosense Webster, Inc. Dr Pürerfellner has received consulting fees from Biosense Webster, Inc, Abbott, Boston Scientific, Biotronik, and Medtronic, and has received payment or honoraria for lectures or presentations from Biosense Webster, Inc, Abbott, Boston Scientific, Biotronik, and Medtronic. Dr Jais has received research grants from Biosense Webster, Inc; has received speaker fees from Biosense Webster, Inc; is a shareholder of Farapulse/Affera; and has received speaker fees and research grants from Boston Scientific, Medtronic, and Abbott. Dr Reddy is a consultant for Biosense Webster, Inc; and unrelated to this manuscript, he serves as a consultant for and has equity in Ablacon, Acutus Medical, Affera-Medtronic, Apama Medical-Boston Scientific, Anumana, APN Health, Aquaheart, Atacor, Autonomix, Axon Therapies, Backbeat, BioSig, CardiaCare, CardioNXT/AFTx, Circa Scientific, CoRISMA, Corvia Medical, Dinova-Hangzhou DiNovA EP Technology, East End Medical, EPD-Philips, EP Frontiers, Epix Therapeutics-Medtronic, EpiEP, Eximo, Farapulse-Boston Scientific, Field Medical, Focused Therapeutics, HRT, Intershunt, Javelin, Kardium, Keystone Heart, LuxMed, Medlumics, Middlepeak, Neutrace, Nuvera-Biosense Webster, Oracle Health, Restore Medical, Sirona Medical, SoundCath, Valcare; unrelated to this work, has served as a consultant for Abbott, AtriAN, BioTel Heart, Biotronik, Boston Scientific, Cairdac, Cardiofocus, Cardionomic, CoreMap, Fire1, Gore & Associates, Impulse Dynamics, Medtronic, Novartis, Philips, Pulse Biosciences; and has equity in DRS Vascular, Manual Surgical Sciences, Newpace, Nyra Medical, Surecor, and Vizaramed. Johnson & Johnson MedTech has an agreement with the Yale Open Data Access Project to serve as the independent review panel for evaluation of requests for clinical study reports and participant-level data from investigators and physicians for scientific research that will advance medical knowledge and public health. Requests for access to the study data can be submitted through the Yale Open Data Access Project site at http://yoda.yale.edu. The other authors report no conflicts.

### Supplemental Material

Figures S1 and S2

Tables S1–S4

## Supplementary Material

## References

[1] Reddy VY Koruth J Jais P Petru J Timko F Skalsky I Hebeler R Labrousse L Barandon L Kralovec S. Ablation of atrial fibrillation with pulsed electric fields: an ultra-rapid, tissue-selective modality for cardiac ablation. JACC Clin Electrophysiol. 2018;4:987–995. doi: 10.1016/j.jacep.2018.04.005 30139499 10.1016/j.jacep.2018.04.005

[2] Verma A Asivatham SJ Deneke T Castellvi Q Neal RE 2nd. Primer on pulsed electrical field ablation: understanding the benefits and limitations. Circ Arrhythm Electrophysiol. 2021;14:e010086. doi: 10.1161/CIRCEP.121.010086 34538095 10.1161/CIRCEP.121.010086

[3] Yavin H Brem E Zilberman I Shapira-Daniels A Datta K Govari A Anic A Wazni O Anter E. Circular multielectrode pulsed-field ablation catheter Lasso pulsed field ablation: lesion characteristics, durability, and effect on neighboring structures. Circ Arrhythm Electrophysiol. 2021;14:e009229. doi: 10.1161/CIRCEP.120.009229 33417475 10.1161/CIRCEP.120.009229 PMC7909749

[4] Reddy VY Anter E Rackauskas G Peichl P Koruth JS Petru J Funasako M Minami K Natale A Jais P. Lattice-tip focal ablation catheter that toggles between radiofrequency and pulsed field energy to treat atrial fibrillation: a first-in-human trial. Circ Arrhythm Electrophysiol. 2020;13:e008718. doi: 10.1161/CIRCEP.120.008718 32383391 10.1161/CIRCEP.120.008718

[5] Reddy VY Dukkipati SR Neuzil P Anic A Petru J Funasako M Cochet H Minami K Breskovic T Sikiric I. Pulsed field ablation of paroxysmal atrial fibrillation: 1-year outcomes of IMPULSE, PEFCAT, and PEFCAT II. JACC Clin Electrophysiol. 2021;7:614–627. doi: 10.1016/j.jacep.2021.02.014 33933412 10.1016/j.jacep.2021.02.014

[6] Reddy VY Neuzil P Koruth JS Petru J Funosako M Cochet H Sediva L Chovanec M Dukkipati SR Jais P. Pulsed field ablation for pulmonary vein isolation in atrial fibrillation. J Am Coll Cardiol. 2019;74:315–326. doi: 10.1016/j.jacc.2019.04.021 31085321 10.1016/j.jacc.2019.04.021

[7] Reddy VY Peichl P Anter E Rackauskas G Petru J Funasako M Minami K Koruth JS Natale A Jais P. A focal ablation catheter toggling between radiofrequency and pulsed field energy to treat atrial fibrillation. JACC Clin Electrophysiol. 2023;9:1786–1801. doi: 10.1016/j.jacep.2023.04.002 37227340 10.1016/j.jacep.2023.04.002

[8] Turagam MK Neuzil P Schmidt B Reichlin T Neven K Metzner A Hansen J Blaauw Y Maury P Arentz T. Safety and effectiveness of pulsed field ablation to treat atrial fibrillation: one-year outcomes from the MANIFEST-PF registry. Circulation. 2023;148:35–46. doi: 10.1161/CIRCULATIONAHA.123.064959 37199171 10.1161/CIRCULATIONAHA.123.064959

[9] Verma A Haines DE Boersma LV Sood N Natale A Marchlinski FE Calkins H Sanders P Packer DL Kuck KH; PULSED AF Investigators. Pulsed field ablation for the treatment of atrial fibrillation: PULSED AF pivotal trial. Circulation. 2023;147:1422–1432. doi: 10.1161/CIRCULATIONAHA.123.063988 36877118 10.1161/CIRCULATIONAHA.123.063988 PMC10158608

[10] Duytschaever M De Potter T Grimaldi M Anic A Vijgen J Neuzil P Van Herendael H Verma A Skanes A Scherr D; inspIRE Trial Investigators. Paroxysmal atrial fibrillation ablation using a novel variable-loop biphasic pulsed field ablation catheter integrated with a 3-dimensional mapping system: 1-year outcomes of the multicenter inspIRE study. Circ Arrhythm Electrophysiol. 2023;16:e011780. doi: 10.1161/CIRCEP.122.011780 36735937 10.1161/CIRCEP.122.011780 PMC10026968

[11] Duytschaever M Vijgen J De Potter T Scherr D Van Herendael H Knecht S Kobza R Berte B Sandgaard N Albenque JP. Standardized pulmonary vein isolation workflow to enclose veins with contiguous lesions: the multicentre VISTAX trial. Europace. 2020;22:1645–1652. doi: 10.1093/europace/euaa157 32879974 10.1093/europace/euaa157

[12] Di Biase L Monir G Melby D Tabereaux P Natale A Manyam H Athill C Delaughter C Patel A Gentlesk P; SURPOINT Postapproval Trial Investigators. Composite index tagging for PVI in paroxysmal AF: a prospective, multicenter postapproval study. JACC Clin Electrophysiol. 2022;8:1077–1089. doi: 10.1016/j.jacep.2022.06.007 36137711 10.1016/j.jacep.2022.06.007

[13] Reddy VY Gerstenfeld EP Natale A Whang W Cuoco FA Patel C Mountantonakis SE Gibson DN Harding JD Ellis CR; ADVENT Investigators. Pulsed field or conventional thermal ablation for paroxysmal atrial fibrillation. N Engl J Med. 2023;389:1660–1671. doi: 10.1056/NEJMoa2307291 37634148 10.1056/NEJMoa2307291

[14] Osorio J Hussein AA Delaughter MC Monir G Natale A Dukkipati S Oza S Daoud E Di Biase L Mansour M; Q-FFICIENCY Trial Investigators. Very high-power short-duration, temperature-controlled radiofrequency ablation in paroxysmal atrial fibrillation: the prospective multicenter Q-FFICIENCY trial. JACC Clin Electrophysiol. 2023;9:468–480. doi: 10.1016/j.jacep.2022.10.019 36752484 10.1016/j.jacep.2022.10.019

[15] Schmidt B Bordignon S Neven K Reichlin T Blaauw Y Hansen J Adelino R Ouss A Futing A Roten L. EUropean real-world outcomes with Pulsed field ablatiOn in patients with symptomatic atRIAl fibrillation: lessons from the multi-centre EU-PORIA registry. Europace. 2023;25:euad185. doi: 10.1093/europace/euad185 37379528 10.1093/europace/euad185 PMC10320231

[16] Howard B Verma A Tzou WS Mattison L Kos B Miklavcic D Onal B Stewart MT Sigg DC. Effects of electrode-tissue proximity on cardiac lesion formation using pulsed field ablation. Circ Arrhythm Electrophysiol. 2022;15:e011110. doi: 10.1161/CIRCEP.122.011110 36166690 10.1161/CIRCEP.122.011110 PMC9584049

[17] Hsu JC Banker RS Gibson DN Gomez T Berman D Datta K Chen Q Doshi SK. Comprehensive dose-response study of pulsed field ablation using a circular catheter compared with radiofrequency ablation for pulmonary vein isolation: a preclinical study. Heart Rhythm O2. 2023;4:662–667. doi: 10.1016/j.hroo.2023.09.005 37936668 10.1016/j.hroo.2023.09.005 PMC10626186

[18] Yavin HD Higuchi K Sroubek J Younis A Zilberman I Anter E. Pulsed-field ablation in ventricular myocardium using a focal catheter: the impact of application repetition on lesion dimensions. Circ Arrhythm Electrophysiol. 2021;14:e010375. doi: 10.1161/CIRCEP.121.010375 34459210 10.1161/CIRCEP.121.010375

[19] Grimaldi M Quadrini F Caporusso N Troisi F Vitulano N Delmonte V Di Monaco A. Deep sedation protocol during atrial fibrillation ablation using a novel variable-loop biphasic pulsed field ablation catheter. Europace. 2023;25:euad222. doi: 10.1093/europace/euad222 37470452 10.1093/europace/euad222 PMC10434733

