# Supplementary material for: Predictors of Success for Pulmonary Vein Isolation With Pulsed-field Ablation Using a Variable-loop Catheter With 3D Mapping Integration: Complete 12-month Outcomes From inspIRE
Source: Circ Arrhythm Electrophysiol. 2024 Apr 24;17(5):e012667. doi: 10.1161/CIRCEP.123.012667 (PMC11111320; doi:10.1161/CIRCEP.123.012667)
Supplement: Supplementary file 1 [file hae-17-e012667-s001.pdf]

SUPPLEMENTAL MATERIAL

Table S1. Study sites and investigators that enrolled patients who were treated with the VLCC.

Sites	Investigators
AZ Sint Jan	**Mattias Duytschaever**<br>Sebastien Knecht
Centre Hospitalier Universitaire (CHU) de Bordeaux	**Pierre Jais**
London Health Sciences Centre - University Hospital	**Allan Skanes**
Medical University Graz	**Daniel Scherr**
Na Homolce Hospital	**Petr Neuzil**<br>Vivek Reddy<br>Jan Petru
Onze-Lieve-Vrouwziekenhuis Hospital	**Tom De Potter**
Ordensklinikum Linz GmbH / Elisabethinen	**Helmut Pürerfellner**<br>Georgios Kollias
Ospedale Generale Regionale "F. Miulli"	**Massimo Grimaldi**<br>Luigi Di Biase
Southlake Regional Health Centre	**Atul Verma**
University Hospital Center Split	**Ante Anic**
Vilnius University	**Gediminas Rackauskas**<br>Peter Lukac<br>Jim Hansen<br>Andrea Natale
Virga Jessa Ziekenhuis	**Johan Vijgen**<br>Thomas Phlips
Ziekenhuis Oost Limburg	**Hugo Van Herendael**

Table S2. Participant inclusion and exclusion criteria.

Inclusion criteria:<br><br>To participate in the study, candidates were required to meet ALL criteria.	1. Diagnosed with Symptomatic PAF.<br>2. Selected for AF ablation procedure by PVI.<br>3. Failed at least one AAD (class I to IV) as evidenced by recurrent symptomatic AF, or intolerable or contraindicated to the AAD.<br>4. Age 18-75 years.<br>5. Willing and capable of providing consent.<br>6. Able and willing to comply with all pre-, post- and follow-up testing and requirements.
Exclusion criteria:<br><br>Candidates were excluded if ANY of the criteria applied.	1. AF secondary to electrolyte imbalance, thyroid disease, or reversible or non-cardiac cause.<br>2. Previous LA ablation or surgery.<br>3. Patients known to require ablation outside the PV region (e.g., Cavotricuspid Isthmus region, atrioventricular reentrant tachycardia, atrioventricular nodal reentry tachycardia, atrial tachycardia, ventricular tachycardia and Wolff-Parkinson-White).<br>4. Previously diagnosed with persistent AF (> 7 days in duration).<br>5. Severe dilatation of the LA (Left Atrium) >50mm antero-posterior diameter in case of Transthoracic Echocardiography).<br>6. Presence of LA thrombus.<br>7. Severely compromised left ventricular ejection fraction (LVEF) (LVEF <40%).<br>8. Uncontrolled heart failure or New York Heart Association Class III or IV.<br>9. History of blood clotting, bleeding abnormalities or contraindication to anticoagulation (heparin, warfarin, or dabigatran).<br>10. History of a documented thromboembolic event (including TIA) within the past 6 months.<br>11. Previous Percutaneous coronary intervention / myocardial infarction within the past 2 months.

	12. Coronary Artery Bypass Grafting in conjunction with valvular surgery, cardiac surgery (e.g. ventriculotomy, atriotomy) or valvular cardiac (surgical or percutaneous) procedure.<br>13. Unstable angina pectoris within the past 6 months.<br>14. Anticipated cardiac transplantation, cardiac surgery, or other major surgery within the next 12 months.<br>15. Significant pulmonary disease (e.g., restrictive pulmonary disease, constrictive or chronic obstructive pulmonary disease) or any other disease or malfunction of the lungs or respiratory system that produces severe chronic symptoms.<br>16. Known significant PV anomaly that in the opinion of the investigator would preclude enrollment in this study.<br>17. Has known pulmonary vein stenosis.<br>18. Acute illness, active systemic infection, or sepsis.<br>19. Presence of intracardiac thrombus, myxoma, tumor, interatrial baffle or patch or other abnormality that precludes catheter introduction or manipulation.<br>20. Severe mitral regurgitation.<br>21. Presence of implanted pacemaker or Implantable Cardioverter-Defibrillator or other implanted metal cardiac device that may interfere with the IRE energy field.<br>22. Presence of a condition that precludes vascular access (such as inferior vena cava filter).<br>23. Significant congenital anomaly or a medical problem that in the opinion of the investigator would preclude enrollment in this study.<br>24. Categorized as vulnerable population and requires special treatment with respect to safeguards of well-being.<br>25. Current enrollment in an investigational study evaluating another device or drug.<br>26. Women who are pregnant (as evidenced by pregnancy test if pre-menopausal), lactating, or who are of childbearing age and plan on becoming pregnant during the course of the clinical investigation.<br>27. Life expectancy less than 12 months.

	28. Presenting contra-indications for the devices used in the study, as indicated in the respective IFU.<br><br>Additional exclusion criteria for Wave I subjects:<br>29. Contraindication for MRI such as use of contrast agents due to advanced renal disease, claustrophobia etc. (at principal investigator discretion).<br>30. Presence of iron-containing metal fragments in the body.<br>31. Unresolved pre-existing neurological deficit.<br>32. Uncontrolled significant Gastroesophageal Reflux Disease.

Table S3. Univariable and multivariable analysis of primary effectiveness endpoint (Wave II main study per protocol analysis set, N=186).

	Univariable analysis		Multivariable analysis	
**Variables**	**N [1]**	**OR (95% CI)**	**N [1]**	**OR (95% CI)**
Sex (Male vs female)	184	0.78 (0.38, 1.61)		
Age (≥50 vs <50 years)	184	2.02 (0.73, 5.58)	176	1.73 (0.61, 4.89)
BMI category (obese vs normal)	184	0.63 (0.26, 1.50)		
BMI category (overweight vs normal)	184	0.56 (0.26, 1.22)		
Documented symptomatic PAF episodes in the past 12 months (yes vs no)	184	0.86 (0.34, 2.20)		
Average duration of each PAF episode in the past 12 months (≥3 vs <3 hours)	176	1.65 (0.82, 3.33)	176	1.24 (0.69, 2.23)
Symptomatic AF episodes in the past 12 Months (≥9 vs <9)	177	0.96 (0.48, 1.89)		
Has any known cardiovascular medical history (yes vs no)	184	1.54 (0.75, 3.15)		
Congestive heart failure (yes vs no)	184	3.19 (0.44, 23.30)		
Vascular disease (yes vs no)	184	1.03 (0.10, 10.16)		
Hypertension (systemic) (yes vs no)	184	1.75 (0.89, 3.45)	176	1.68 (1.09, 2.59)
Obstructive sleep apnea (yes vs no)	182	0.77 (0.16, 3.79)		
Diabetes (yes vs no)	184	2.52 (0.82, 7.70)	176	2.11 (0.7, 6.36)
CHA2DS2-VASc score (>2 vs ≤2)	184	1.63 (0.72, 3.68)		
LVEF (≥60 vs <60%)	184	0.27 (0.13, 0.55)	176	0.27 (0.16, 0.48)
LA diameter (mm) (≥39 vs <39 mm)	184	1.04 (0.53, 2.05)		
LA volume (≥34 vs <34 mL/m2)	113	1.28 (0.50, 3.26)		
AFEQT (≥64 vs <64)	181	0.91 (0.46, 1.79)		
Number of valid PFA applications (≥48 vs <48)	184	0.30 (0.14, 0.66)	176	0.25 (0.13, 0.49)
Total ablation duration (≥22 vs <22 min)	184	1.61 (0.81, 3.19)		
Total procedure time (≥61 vs <61 min)	184	1.72 (0.86, 3.42)		
Total pulsed field ablation time with circular IRE catheter (≥22 vs <22 min)	184	1.57 (0.79, 3.10)		
Sedation method (general anesthesia vs conscious sedation)	184	0.46 (0.23, 0.93)	176	1.02 (0.58, 1.78)

AF indicates atrial fibrillation; AFEQT, AF effect on quality of life; BMI, body mass index;

CHA_2DS_2-VASc, Congestive heart failure, Hypertension, Age ≥75 years (doubled), Diabetes mellitus, prior Stroke or TIA or thromboembolism (doubled), Vascular disease, Age 65 to 74 years, Sex category; IRE, irreversible electroporation; LA, left atrium; LVEF, left ventricular ejection fraction; PAF, paroxysmal atrial fibrillation; PFA, pulsed field ablation.

Table S4. Comparison between procedures performed under general anesthesia and sedation (Wave II, N=186).

Variable	Sedation (N=54)	General Anesthesia (N=132)	p-value
Total procedure time (min)	73.07±20.49	68.83±30.08	0.015
Total fluoroscopy time (min)	8.07±5.35	7.75±7.58	0.036
Total catheter LA dwell time (min)	46.74±19.50	43.86±21.11	0.121
Total LA mapping time (min)	6.69±3.31	7.68±4.44	0.265

Kruskal Wallis test. SD, Standard Deviation; LA, left atrium.

Figure S1. Effectiveness analysis in the per-protocol population, showing 12-month freedom from A) asymptomatic/symptomatic recurrences with or without protocol-driven remote rhythm monitoring and (B) symptomatic recurrences with or without protocol-driven remote rhythm monitoring.

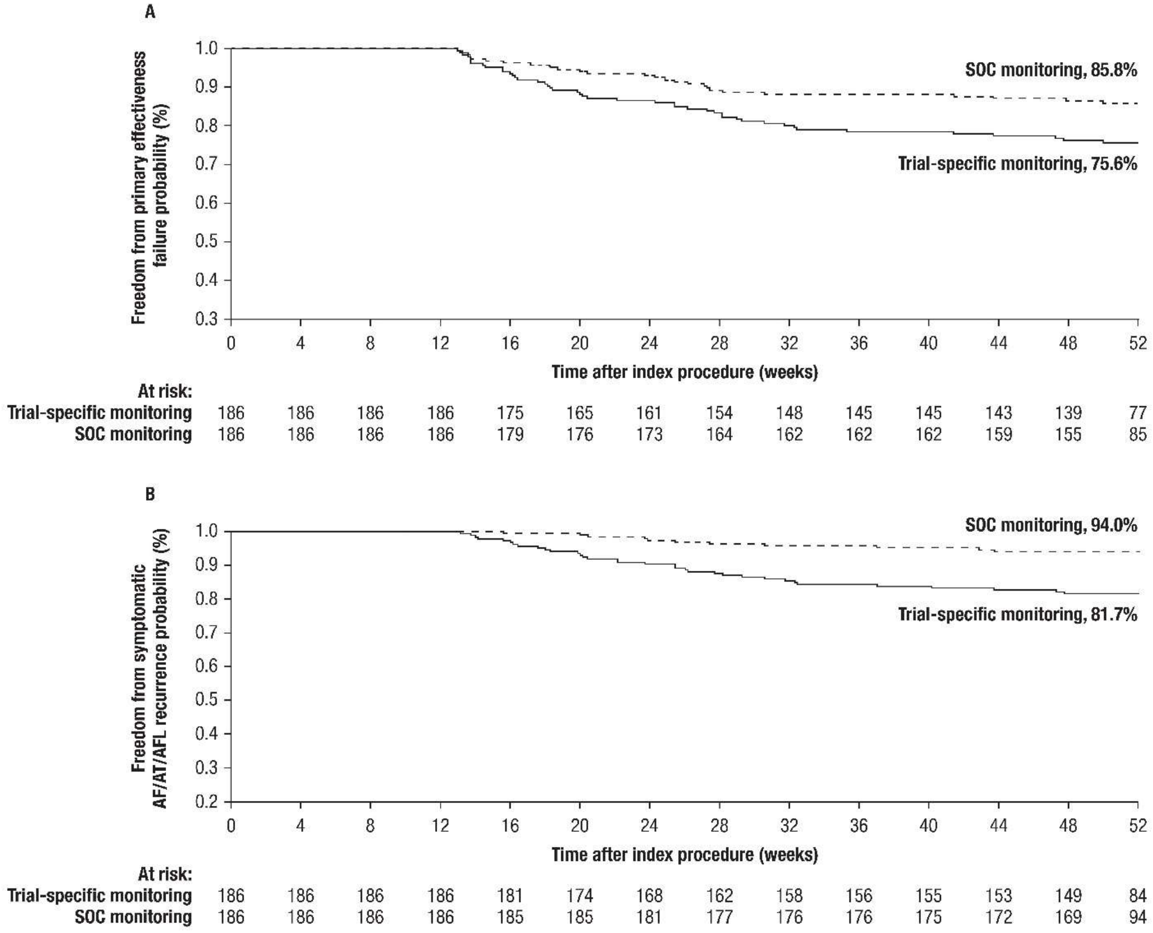

AF indicates atrial fibrillation; AFL, atrial flutter; AT, atrial tachycardia; SOC, standard-of-care.

Figure S2. Forest plot of multivariable analysis of primary effectiveness endpoint using GEE (Wave II main study per protocol analysis set, N=186).

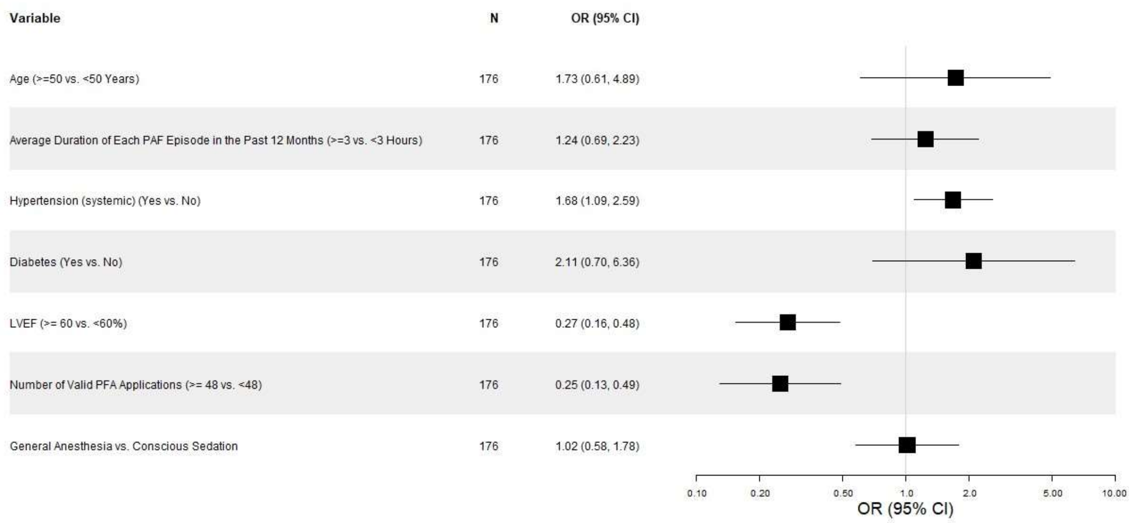

GEE indicates generalized estimating equation; LVEF, left ventricular ejection fraction; PAF, paroxysmal atrial fibrillation; PFA, pulsed field ablation.
